# Photochemistry and Cytotoxicity Evaluation of Heptamethinecyanine Near Infrared (NIR) Dyes

**DOI:** 10.3390/ijms140918557

**Published:** 2013-09-09

**Authors:** David S. Conceição, Diana P. Ferreira, Luís F. Vieira Ferreira

**Affiliations:** Molecular Physical Chemistry Center, and IN-Institute of Nanoscience and Nanotechnology, Instituto Superior Técnico, Technical University of Lisbon, Av. Rovisco Pais, 1049-001 Lisboa, Portugal; E-Mails: david.conceicao@ist.utl.pt (D.S.C.); diana.ferreira@ist.utl.pt (D.P.F.)

**Keywords:** heptamethine cyanine dyes, fluorescence quantum yields and lifetimes, transient absorption, PDT, HeLa cells, photo and dark cytotoxicity, confocal microscopy

## Abstract

The present study investigates the photochemical properties of potential photosensitizers for photodynamic therapy, namely four commercial heptamethinecyanine dyes (IR125, IR780, IR813, IR820). Spectroscopic studies were made by means of laser induced fluorescence and laser flash photolysis in order to obtain fluorescence quantum yields and transient absorption spectra. Fluorescence lifetimes were also determined. The differences encountered were essentially related with the interaction of the sulfonate groups with the solvent, and also regarding the rigidification of the central bridge connecting the two nitrogen-containing heterocyclic groups. Transient absorption studies were performed both in aerated and oxygen free samples, to conclude about the formation of photoisomers and triplet state. For the four dyes under study, a cytotoxic evaluation in the dark and after irradiation was performed using HeLa cells as the model cell line, which revealed significant changes after irradiation mainly in IR125 and IR813 dyes. Confocal microscopy analysis showed that these dyes tend to enter to the intracellular space.

## 1. Introduction

Photodynamic therapy (PDT), is a form of phototherapy using non-toxic light-sensitive compounds, which, when exposed selectively to light, become toxic to targeted malignant and other diseased cells. This therapy is based on the use of a chemical compound activated by light of a specific wavelength. Four important factors are involved in the effects of photodynamic therapy: the target tissue, oxygen intermediates, light source and photosensitizers [1].

**Figure 1:**
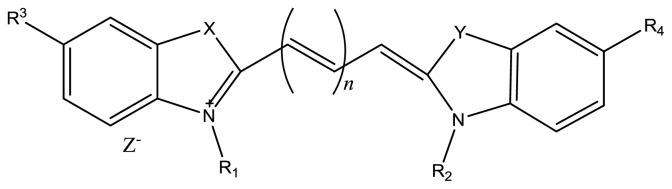
General structure of polymethine cyanine dyes.

Regarding the photosensitizer, certain features must be achieved to enable a responsive phototherapy, for instance, high absorption at longer wavelengths, in which tissue light scattering is reduced allowing deeper penetration of light into tissues (600–900 nm), high quantum yield for the production of singlet oxygen, high chemical stability, and low dark toxicity. Taking this into account, near infrared (NIR) dyes have been the subject of innumerous studies concerning their application in several fields of interest as fluorescent markers but also as therapeutic agents in PDT [2,3]. The advantages of using NIR dyes include minimal interfering absorption and fluorescence from biological samples, reduced scattering and enhanced tissue penetration depth [4,5]. The most common NIR dyes are cyanine dyes with two aromatic nitrogen-containing heterocycles linked by a polymethine bridge (Figure 1). In clinical practice, the most used are pentamethine and heptamethine cyanines composed of benzoxazole, indole, and quinolone [6,7]. Their spectral properties can be tuned by the appropriate choice of heterocyclic nuclei (X and Y), by the length of the polymethine chain (*n*), and also by varying the groups R_1_–R_4_ for instance, to provide the desired aqueous solubility.

**Figure 2:**
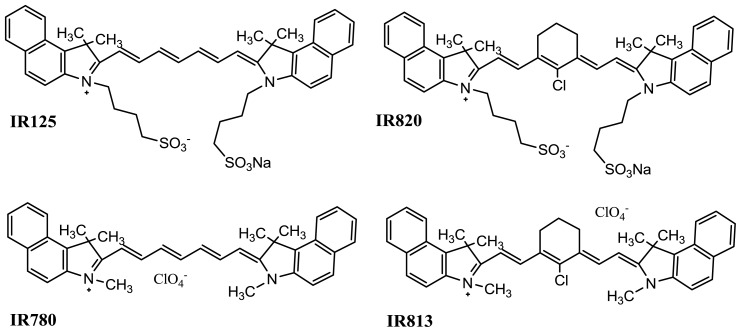
Structure of the photosensitizers used in this work.

In this work, two classes of cyanine dyes were studied: di-benzo-indotricarbocyanines rigidified and non-rigidified. The rigidification in these dyes is achieved with the inclusion of a chloro-cyclohexenyl ring within the polymethine bridge (Figure 2). The purpose was initially to analyze the effect of the rigidification and sulfonate groups on the fluorescence emission and on the triplet state by means of laser induced fluorescence and laser flash photolysis respectively, and to make conclusions about the possible formation of photoisomers, and residual photodegradation products. Cyanine dyes in the excited singlet state can decay back to the ground state through four major pathways: fluorescence, intersystem crossing, internal conversion, and photoisomerization. Of the radiationless decay processes, it has been suggested that photoisomerization is the most significant followed by internal conversion [8–10]. The level of photoisomerization depends on dye rigidity, so, by introducing rigidifying structures or rotation-hindering bulky substituents, photoisomerization would be expected to decrease with a corresponding increase in quantum yield.

Biological assays were also performed using HeLa cells, a cellular line derived from cervical cancer cells. The goal was to evaluate the dark cytotoxic effect of each dye in the cellular culture and also their phototoxicity after irradiating the cells with a 250 W quartz tungsten-halogen lamp. The 3-(4,5-dimethylthiazol-2-yl)-2,5-diphenyltetrazolium bromide (MTT) assay was a useful protocol in these conditions, to characterize the intracellular activity, regarding some mitochondrial dehydrogenases, which would be proportional to the total amount of formazan formed in the intracellular enzymatic reaction [11]. This could enable a better comprehension about the generation of intracellular reactive oxygen species (ROS) or toxic decomposition photoproducts, the key of cell destruction in photodynamic therapy of cancer.

One of the main challenges of IR125 is its poor stability in aqueous solution and fast degradation rates [12], and thus, one would also expect to see some improvements *in vitro* and in solution, when rigidifying the central ring. Transient absorption spectra present useful information concerning residual photoproducts, which can be correlated with the cytotoxic assays.

## 2. Results and Discussion

### 2.1. Ground State UV-Visible Spectral Characterization

**Figure 3:**
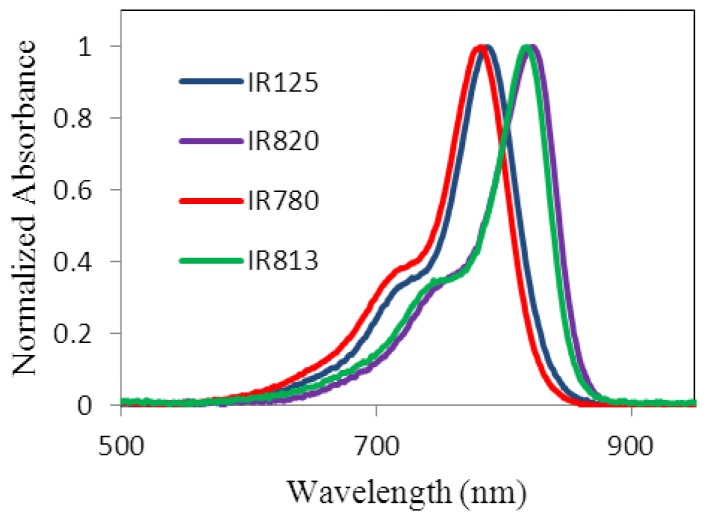
Absorption spectra of all dyes, in ethanol.

The ground state absorption spectra of all dyes in ethanol are presented in Figure 3 and also the λ_max_ for each dye are indicated in Table 1. The non-rigidified dyes exhibit a maximum at 777 nm and 783 nm, while the rigidified have a maximum peak at about 815 nm and 818 nm. The bathocromic shift observed between the non-rigidified and the ones rigidified with a cyclohexenyl ring within the polymethine bridge, exhibits the effect of the structural modification, which promotes a resonance increase and therefore absorption at longer wavelengths. The inclusion of the sulfonate groups in the end of the alkyl chain can improve the solubility in aqueous media and can also prevent the dye from aggregating [13]. In addition, for the sulfonate groups, the hydrogen bonds formed in ethanol act as an anchor that certainly decreases the non-radioactive pathways of deactivation such as photoisomerization. Resonance implies planarity; therefore, all planar spatio-configurations of the molecule lead to an increase of the overall electron resonance and increase the conjugated system. All these effects deeply affect the absorption wavelength and as a final result, the absorption spectra of the dye in ethanol shifts to lower energies compared with the non-sulfonated dyes [14].

It is well known that these dyes have a high probability of forming aggregates influencing their optical properties [15,16]. Therefore, aggregation studies were performed in the concentration range from 1.5 × 10^−5^ M to 3.0 × 10^−7^ M, in ethanol. No aggregates were detected in the concentration range under study.

### 2.2. Fluorescence Emission Spectra and Fluorescence Lifetime Decays

**Figure 4:**
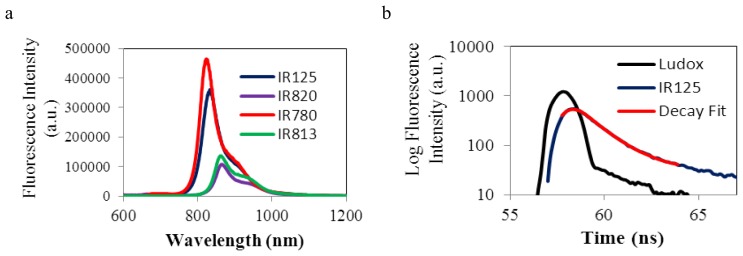
(**a**) Fluorescence emission spectra of all dyes, in ethanol; (**b**) Fluorescence lifetime decay of IR125, in ethanol.

Rigidifying chromophore systems in organic molecules is an established strategy to minimize the non-radioactive pathways of deactivation, therefore to improve fluorescence quantum yields (Φ_F_) and fluorescence lifetimes (τ_F_) [17]. Several reports have demonstrated that incorporating a cyclohexenyl ring in the polymethine chain minimizes non-radiative decay via *trans*-*cis* isomerization, thereby increasing the quantum yield and the fluorescence lifetimes [18]. However, as it can be verified from the data and spectra obtained in Table 1 and Figure 4 respectively, the rigidification of the polymethine bridge did not enhance both fluorescence quantum yield and fluorescence lifetimes. In fact, a significant decrease was detected when comparing IR125 with IR820, and IR780 with IR813, evidencing a greater loss of excited energy through non-radiative pathways. The most probable cause must be the heavy atom effect caused by the Cl atom situated in the cyclohexenyl ring [19,20]. It was said in the previous section that resonance requires planarity. Since the rigidified dyes absorb at longer wavelengths it can be concluded that the introduction of the cyclohexenyl ring made the molecules more planar, in comparison with the non-rigidified. However, by analyzing the Φ_F_ values, it can be observed that the rigidified dyes have the lowest fluorescence quantum yield values, denoting that the loss of excited state energy was most probably due to the rate of intersystem crossing to the triplet state. Also, as mentioned before, the sulfonate groups of dyes IR125 and IR820 seem to constrain the spatio-configuration of the molecule, due to the formation of hydrogen bonds with the alcoholic solvent. But, while these bonds make the molecule more planar, another important issue must be discussed, taking into account the decrease of the Φ_F_ value, from the non-sulfonated to the sulfonated dyes. The methyl groups of dyes IR780 and IR813 have higher electron donating capacity by induction than the methylene groups of dyes IR125 and IR820 and therefore these non-sulfonated lead to higher fluorescence quantum yields.

### 2.3. Transient Absorption Studies

**Figure 5:**
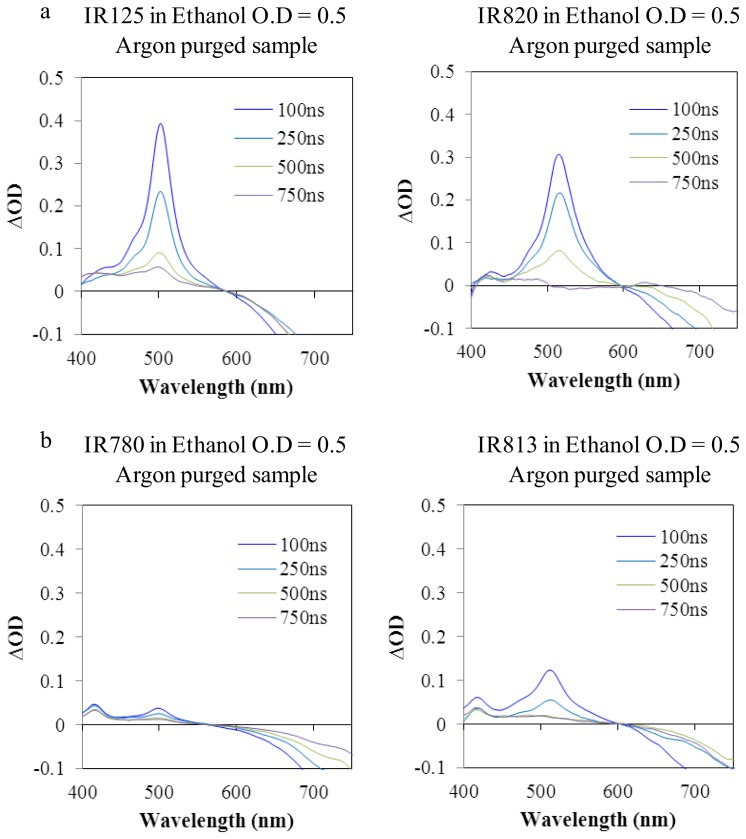
Transient absorption spectra of argon purged dyes, prepared at an optical density of 0.5, in ethanol. (**a**) Sulfonated dyes; (**b**) Non-sulfonated dyes.

In this work, and due to the detection limits of our equipment (0.01), it was not possible to evaluate the singlet oxygen quantum yields of the dyes under study confirming that these cyanines are weak generators of ^1^O_2_ (a common property for several cyanines) [21–23]. Cyanines are characterized by a truly complex pattern of photophysical properties. We already confirm that the fluorescence quantum yields in this work are relatively low (less than 0.17) and no singlet oxygen formation was detected suggesting that the quantum yields are also low. Thus, we evaluated the possible occurrence of non-radiative deactivation pathways (photoisomerization), the generation of photoproducts and also the intersystem crossing to the triplet state with the use of transient absorption laser flash photolysis [24]. From the transient absorption spectra (Figure 5), the ground state depletion could easily be identified, but another two different signals arose at approximately 415 nm and 500 nm, in non-sulfonated dyes (IR780 and IR813), which could be the triplet state or the formation of the photoisomer of the cyanines under study. No triplet state and photoisomer signals were detected in air equilibrated samples (data not shown).

**Figure 6:**
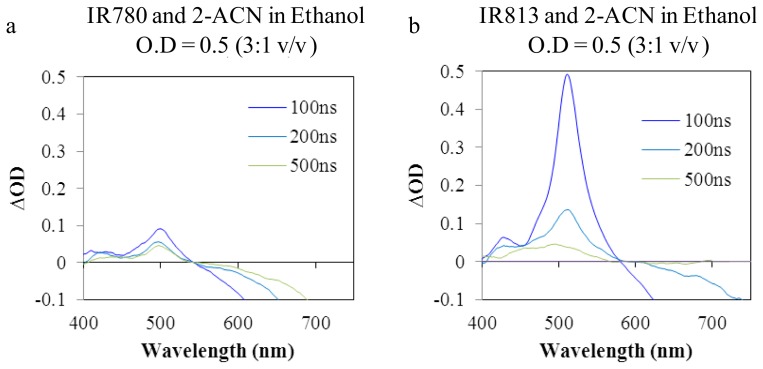
Transient absorption spectra of (**a**) IR813 and (**b**) IR780, in ethanol, sensitized by 2-acetonaphtone, in argon purged samples.

In order to identify the triplet state, a triplet-triplet sensitizer (2-acetonaphtone) was used. Indeed, the signal was located at about 500nm, in argon purged samples, as the results presented in Figure 6 show. Triplet energy transfer from 2-acetonaphtone to the dye was also studied, resulting in an increase of the initial triplet state signal. Figure 5a shows the transient spectra of the sulfonated dyes (IR125 and IR820) were the formation of the photoisomer is not visible at ~415nm. Thus, the functionalization of the dyes with the sulfonate groups could effectively lead to the formation of hydrogen bonds with the solvent stabilizing the structure of the dye and preventing the torsional rotation which ultimately leads to photoisomerization.

The generation of photoproducts could also be addressed by this technique, by analyzing the triplet decay presented in Figure 5. The triplet state signal of IR820 decayed approximately to zero, at 750 ns, suggesting that there were no major photoproducts formed. On the contrary, IR125 and IR813 still have some significant residual signal, evidencing the presence of photoproducts generated after laser excitation. These photoproducts could be potentially toxic, regarding the dyes inclusion in cells for *in vitro* cellular studies, as it will be addressed in the following section.

### 2.4. Cytotoxicity Studies

**Figure 7:**
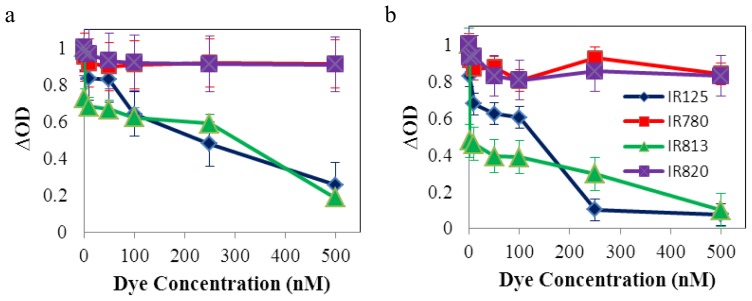
*In vitro* cytotoxicity of all dyes prepared with six different concentrations values, in dimethyl sulfoxide (DMSO). (**a**) Optical density of produced formazan, in the dark and (**b**) after irradiation.

As it was described in an earlier section, all dyes were tested *in vitro* in HeLa cells, in the absence and presence of light. Six different concentrations of the dyes in dimethyl sulfoxide (DMSO) were tested, in the range of the nanomolar, to analyze the behavior of the cell culture at very low concentrations, and the results were obtained relatively to the optical density of formazan formed in the different conditions (Figure 7). The maximum tolerable amount of DMSO in culture was tested, being 1% a value that maintained the cell culture with no significant toxic effects (data not shown). Wells with high intracellular activity, concerning the mitochondrial dehydrogenases related with the MTT assay, would also have higher values of optical density and a lower cytotoxic effect, since formazan is an enzymatic intracellular product that only stains living and metabolically active cells. Therefore, to obtain this short-term cytotoxic effect, in each condition, the following formula was used:

(1) $$\text{Cytotoxicity }(\%)=1-\frac{\text{OD of Test well}-\text{OD of Blank}}{\text{OD of control well}-\text{OD of Blank}}\times 100$$

where the test well constrained the dye within the cellular culture, the control well referred to those wells without any dye, and the blank designated a single well in each 96-well plate with only MTT solution.

**Figure 8:**
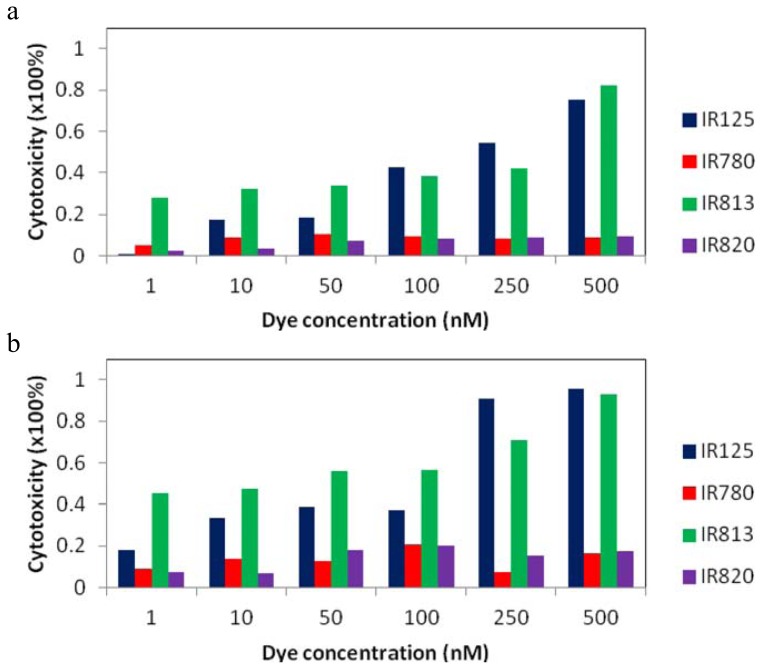
*In vitro* cytotoxicity after inoculation with IR125, IR780, IR813 and IR820, at six different concentrations: 1 nM, 10 nM, 50 nM, 100 nM, 250 nM and 500 nM. (**a**) Dark cytotoxicity; (**b**) Photo cytotoxicity.

As it can be observed in Figure 8, the most cytotoxic dyes are IR125 and IR813. At 500 nM, the first one exhibited a dark cytotoxicity of 75% and 95% after irradiation, whereas the latter one revealed a value of 82% in the dark and 93% after irradiating the plate. It is known that following intersystem crossing to the triplet state, the sensitizer can interact with molecular oxygen via a triplet-triplet annihilation process, generating singlet oxygen (Type II reaction), or, alternatively, the sensitizer in its triplet state can participate in electron transfer processes or radical intermediate formation, also leading to the generation of phototoxic species such as O_2_, HO_2_·, HO_2_^−^, H_2_O_2_ and HO·[25]. However, and taking into account the concentrations used in this work, no significant effects should be observed in the presence of light to damage the cellular constituents [26,27]. Thus, the main cause for the significant values of cytotoxicity presented by IR125 and IR813 should be related with their instability in solution (during long periods of time), and degradation products and photoproducts that arose during the inoculation of the dyes in the cellular culture. It seems though that the rigidification of dye IR125 with a cyclohexenyl ring promoted a significant molecular stabilization, since IR820 exhibited no major cytotoxic effects, both in the presence and absence of light. However, and not as expected, the exact opposite was observed for dyes IR780 and IR813. The rigidification showed a remarkable increase of the dye’s toxicity, evidencing that the stability of the molecule was greatly affected by the introduction of the ring within the polymethine bridge. However, these results can be correlated with those obtained from transient absorption studies, which revealed, as it was already discussed, that IR125 and IR813 had significant residual photoproducts. These remaining degradation products could be a strong cause for the high cytotoxic values presented by these two dyes.

### 2.5. Confocal Fluorescence Microscopy

**Figure 9:**
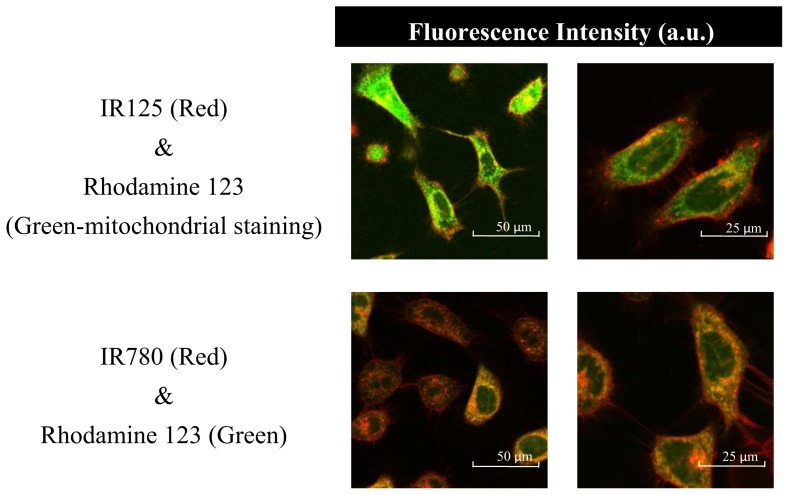
Confocal fluorescence microscopy analysis: co-localization of dyes IR125 and IR780 with mitochondrial-specific tracker, Rhodamine 123, in cultured HeLa cells.

A confocal microscopy analysis was also attempted to study the localization of the dyes when inoculated with HeLa cell culture. For that purpose, a sulfonated dye with extremely high cytotoxic effects in these conditions, such as dye IR125 was compared to a non-sulfonated dye with low cytotoxicity, IR780. The results, illustrated in Figure 9, show that both dyes easily enter to the intracellular microenvironment, with some suggestion of a mitochondrial localization pattern, and even to the nucleous, immediately discarding any chance of the dye being accumulated only on the extracellular matrix and not entering inside cells. In photodynamic therapy, nuclear photodamage is generally considered to be a potentially adverse effect since mutagenic results might result and therefore, from this point of view, these dyes do not seem to be good candidates for that purpose. The sulfonate groups of dye IR125 did not reveal any specific effect regarding its efficiency *in vitro*, when compared with IR780.

## 3. Experimental Section

### 3.1. Materials

The four heptamethinecyanine dyes studied in this work were purchased from Sigma-Aldrich and used without further purification. Ethanol (Merck, Uvasol grade, Darmstadt, Germany) was used as received. Fetal bovine serum (FBS), l-glutamine, penicillin and streptomycin, phosphate buffer saline (PBS), MTT powder, and potassium dichromate were all acquired from Sigma-Aldrich. PBS was diluted 10× with de-ionized water before usage, and MTT was prepared in PBS, at 5 mg/mL. Dulbecco’s Modified Eagle’s Medium (DMEM) was purchased from Invitrogen.

### 3.2. UV-Visible Absorption Spectra

The steady-state absorption spectra was recorded with the use of a Camspec M501 single beam scanning UV/visible spectrophotometer at room temperature and in the spectral range of 200 to 1000 nm. All the solutions were prepared in ethanol and adjusted to an absorbance of 0.6 at 337 nm using a UV quartz cuvette (1 cm path length).

### 3.3. Laser-Induced Luminescence: Fluorescence Emission Quantum Yield Determinations

The schematic diagrams for the Laser induced luminescence system are presented in reference [28,29]. A N_2_ laser (PTI model 2000, *ca.* 600 ps FWHM, ~1.0 mJ per pulse) was used, with an excitation wavelength of 337 nm. With this set-up, luminescence spectra were easily acquired by the use of an InGaAs photodiode array detection system (PDA) from Andor, model i-Dus, working at −60 °C. This detector allows the acquisition of signals that arise in the NIR range (700 nm to 1700 nm), and that are not possible to acquire using a normal UV-visible detector. The light arising from the irradiation of the samples by the laser pulse was collected by a collimating beam probe coupled to an optical fiber (fused silica). The PDA was coupled to a fixed compact imaging spectrograph (Andor, model Shamrock 163). Corrected spectra were obtained by applying a calibration curve to the range already specified (700 nm to 1700 nm). The fluorescence quantum yields of each of the cyanine dyes were calculated relative to the standard from their respective average fluorescence peak areas, and the published quantum yield of the standard, which in this case was HITC (1,3,3,1′,3′,3′,-hexamethyl-2,2′-indotricarbocyanine iodide) dye (Φ_F_ = 0.28 in ethanol). The quantum yield measurements were made with the use of reflection geometry (front face), in order to avoid adverse effects due to the small Stokes shift (therefore to minimize the re-absorption effects). The optical density at the excitation wavelength (337 nm from the N2 laser) used for both the unknown and standard samples was 0.60. The methodology used for determinations of the fluorescence quantum yields in high concentrated solutions was described in detail in reference [30].

### 3.4. Fluorescence Lifetimes Determination

Fluorescence lifetimes were determined using Easylife V^™^ equipment from OBB (Birmingham, NJ, USA) Lifetime range from 90 ps to 3 μs). This technique uses pulsed light sources from different LEDs (630 nm in this case) and measures fluorescence intensity at different time delays after the excitation pulse. In this case a 665 nm cut-off filter was used. The instrument response function was measured using a Ludox scattering solution. FelixGX software from OBB was used for fitting and analysis of the decay dynamics.

### 3.5. Laser Flash Photolysis: Transient Absorption Measurements

The schematic diagrams for the laser flash photolysis technique are presented in references [28,29]. For the laser flash spectra shown, the fourth harmonic of a Nd:YAG laser (266 nm, *ca.* 6 ns full-width half maximum, FWHM) from B. M. Industries (Thomson-CSF, model Saga 12-10, Evry, France), and the transmission mode was employed in this case, the monitoring lamp being a 250 quartz tungsten-halogen in a Oriel housing. The samples were prepared with an optical density of 0.5 at the excitation wavelength, and oxygen removal was achieved by argon bubbling for about 15 min for each sample. Transient absorption data are reported as change of optical density ΔO.D or percentage of absorption defined by:

(2) $$\%\text{Abs}=100\times J_{t}/J_{0}=100\times (1-J_{t}/J_{0})$$

where *J*_0_ and *J*_t_ are the transmitted light from sample before exposure to the exciting laser pulse and at time *t* after excitation, respectively.

### 3.6. HeLa Cell Culture

The HeLa cell line was cultured at 37 °C with 5% CO_2_ in DMEM culture medium containing 10% heat inactivated FBS, 200 mM l-glutamine, 1% penicillin and streptomycin. Cultures at ~90% confluence were routinely split 1:3 in T25 flasks as follows. Initially, cells were washed in PBS. One milliliter (1 mL) of PBS containing 0.25% (*w*/*v*) of trypsin was added to the T25 flask and placed at 37 °C for 10 min. After the cells were detached from the dishes, 1 mL of pre-warmed culture medium was added and the cells transferred to an 18 mL falcon tube. Cells were spun down at 1500 rpm for 5 min and plated in a new T25 flask with fresh culture medium with a concentration of 1.5 × 10^6^ M.

### 3.7. Preparation of Dyes and Inoculation with HeLa Cells

Cells were plated with a concentration of 30,000 cells per well in two 96 Well plate. One of the plates was used for dark cytotoxicity quantification, and the other for photo-cytotoxicity evaluation. After 24 h, IR125, IR780, IR813 and IR820 were added to the wells, in triplicate, with six different concentrations, in DMSO: 1 nM, 10 nM, 50 nM, 100 nM, 250 nM and 500 nM. The concentration of DMSO in the final volume of the well did not exceed 1%. Also, triplicates of control wells (cells incubated without dye) were prepared. Cells were then incubated at 37 °C with 5% CO_2_ for 24 h.

### 3.8. Irradiation of HeLa Cells for Photo-Cytoxicity Evaluation

**Figure 10:**
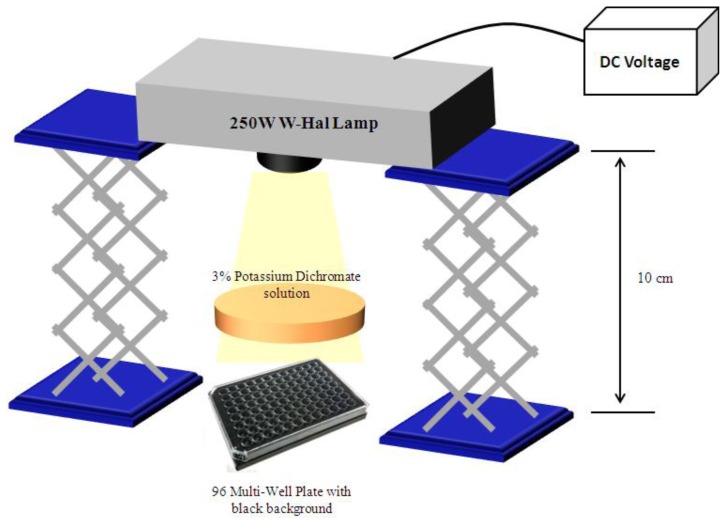
Experimental set-up for photo-cytotoxic evaluation of near infrared (NIR) dyes in HeLa cells.

The 96-well plate prepared for photo-cytoxicity was irradiated with a 250 W Xe lamp, for 15 min, according to Figure 10. The lamp was placed approximately 10 cm above the microplate, and a 3% solution of potassium dichromate was used, to cut wavelengths below 500 nm. The distance was considered “ideal” since the temperature directly measured in the potassium dichromate solution did not exceed the 28 °C. At the absorption bands (between 780 nm and 820 nm), the light dose was approximately 200 mW/m^2^.

### 3.9. Cytotoxicity Assays

To evaluate and quantify the cytotoxicity of the dyes inoculated with the cells, the MTT assay was performed. The media was removed and replaced by 100 μL of MTT working solution at 0.5 mg/mL, in serum and phenol red-free DMEM medium, followed by a four hour incubation at 37 °C with 5% CO_2_. To dilute the formazan crystals formed, the MTT working solution was discarded and replaced by 200 μL of DMSO followed by a 15 min incubation at 37 °C with 5% CO_2_. Using a BMG SPECTROstar Nano plate reader, the absorbance values at 570 nm were recorded, for both the dark cytotoxicity and photo-cytotoxicity plates.

### 3.10. Confocal Fluorescence Microscopy

All measurements were performed on a Leica TCS SP5 (Leica Microsystems, Mannheim, Germany) inverted confocal microscope (DMI6000). A 63× apochromatic water immersion objective with a NA of 1.2 (Zeiss) was used for all experiments, and an Argon laser was used for excitation. Rhodamine 123 was used at 5 μM for mitochondrial staining, whereas the cyanine dyes to test were prepared at 500 nM, in DMSO. A co-staining of Rhodamine 123 with the cyanine dye was performed, and both of them were added to the chamber constraining cells at 2 × 10^5^ cells/chamber, following 30 min incubation at 37 °C. Images were acquired at 100 Hz and sequentially in two different channels, one regarding the mitochondrial staining with Rhodamine 123, excited at 476 nm with 5% of laser intensity, in a detection range from 505 nm to 560 nm, and another channel excited at 633 nm with 50% of laser intensity, referring to the cyanine dye staining, and with a range from 650 nm to 800 nm.

## 4. Conclusions

With these dyes the rigidification of the polymethine bridge did not increase both fluorescence quantum yield and fluorescence lifetimes. In fact, probably due to steric hindrance effects, the torsional rotation of dyes IR813 and IR820 must be higher compared with the non-rigidified dyes, and thus, most of the excited state energy is lost through non-radiative pathways. Transient absorption measurements show different transients in argon purged samples, one being the triplet state (at approximately 490 nm) and another one that seems to “live” even longer, regarding the formation of photodegradation products. In air equilibrated samples no photoisomer was identified. All dyes were then tested *in vitro* with HeLa cells to evaluate the cytotoxic effects of each dye in the presence and absence of light. The main cause for the high cytotoxic values observed for dyes IR125 and IR813 should be their instability in solution and decomposition photoproducts. It seems though that the rigidification diminished the instability of dye IR125, since in the absence and presence of light there was a minor cytotoxic effect of dye IR820, whereas for dye IR813 occurred exactly and surprinsingly the opposite. Confocal microscopy studies showed that both the sulfonated (IR125) and non-sulfonated dye (IR780) enter to the intracellular environment. For successful photodynamic therapy trials though, non-toxic agents that show very substantial effects on viability upon irradiation are needed, and taking into account the data presented in this work, which indicate that the most effective dyes are almost as toxic in the dark as after irradiation, it can be concluded that these agents are not so good candidates to be used as photosensitizers in PDT.

## Figures and Tables

**Table 1:** Spectral properties of the dyes under study, in ethanol.

Dye	Absorption	Emission	Stokes shift	Φ_F_ in ethanol	τ_F_ (ns)	χ^2^

	λ_max_ (nm)	λ_max_ (nm)	nm/cm^−1^			
HITC	-	-	-	0.28	-	-
IR125	783	833	50/767	0.14	0.66	0.94
IR820	818	867	49/691	0.042	0.40	0.90
IR780	777	823	46/720	0.17	0.80	0.98
IR813	815	863	48/682	0.060	0.37	0.94
